# Pre-Processing a Polymer Blend into a Polymer Alloy by KinetiSol Enables Increased Ivacaftor Amorphous Solid Dispersion Drug Loading and Dissolution

**DOI:** 10.3390/biomedicines11051281

**Published:** 2023-04-26

**Authors:** Stephen A. Thompson, Daniel A. Davis, Dave A. Miller, Sandra U. Kucera, Robert O. Williams

**Affiliations:** 1 Molecular Pharmaceutics and Drug Delivery Division, College of Pharmacy, The University of Texas at Austin, 2409 W. University Ave, PHR 4.214, Austin, TX 78712, USA; 2 AustinPx, LLC, 111 W Cooperative Way, Suite 300, Georgetown, TX 78626, USA

**Keywords:** ivacaftor, amorphous solid dispersion, polymer, polymer alloy, drug loading, Kalydeco, dissolution, solubility, hydrogen bonding

## Abstract

This study compares the effects of pre-processing multiple polymers together to form a single-phase polymer alloy prior to amorphous solid dispersion formulation. KinetiSol compounding was used to pre-process a 1:1 (*w*/*w*) ratio of hypromellose acetate succinate and povidone to form a single-phase polymer alloy with unique properties. Ivacaftor amorphous solid dispersions comprising either a polymer, an unprocessed polymer blend, or the polymer alloy were processed by KinetiSol and examined for amorphicity, dissolution performance, physical stability, and molecular interactions. A polymer alloy ivacaftor solid dispersion with a drug loading of 50% *w*/*w* was feasible versus 40% for the other compositions. Dissolution in fasted simulated intestinal fluid revealed that the 40% ivacaftor polymer alloy solid dispersion reached a concentration of 595 µg/mL after 6 h, 33% greater than the equivalent polymer blend dispersion. Fourier transform infrared spectroscopy and solid-state nuclear magnetic resonance revealed changes in the ability of the povidone contained in the polymer alloy to hydrogen bond with the ivacaftor phenolic moiety, explaining the differences in the dissolution performance. This work demonstrates that the creation of polymer alloys from polymer blends is a promising technique that provides the ability to tailor properties of a polymer alloy to maximize the drug loading, dissolution performance, and stability of an ASD.

## 1. Introduction

A substantial number of new chemical entities exhibit poor bioavailability due to the fact of their low water solubility, limiting their therapeutic potential [1]. Based on one estimate, the percentage of new candidates (including preclinical and FDA-approved, as well as discontinued, molecules) that had “high” or “very high” lipophilicity increased from 41% to 54% from the 2001–2010 period to the 2011–2020 period, respectively [2]. One method to improve these compounds’ water solubility is to formulate them as amorphous solid dispersions (ASDs) [3]. In an ASD, the active pharmaceutical ingredient (API) is first blended with the polymer and then processed using a suitable method (e.g., KinetiSol compounding, spray-drying, and hot melt extrusion) to dissolve or disperse the API within the polymer such that the amorphous form is stabilized to preserve its higher solubility [4].

The choice of polymeric carrier can dramatically affect the properties and performance of ASDs. For example, a polymer can alter API–polymer miscibility [5,6] and the physical and chemical stability of the system [7], as well as the dissolution performance of an ASD [8]. Despite the availability of polymers with diverse and useful properties, they also have limitations to consider. For example, while povidone K30 (PVPK30) and copovidone (PVPVA) can have high miscibility with an API that can allow for a high drug loading [9], these polymers also have a high hygroscopicity that can cause physical and chemical instabilities in ASDs [10]. Recent work has examined the ability of hydroxypropyl methylcellulose acetate succinate (HPMCAS) and PVPVA to enhance the dissolution performance of six ASDs containing one of six poorly-soluble APIs [11]. In that study, it was reported that PVPVA-based ASDs exhibited a marked decrease in dissolution performance as a function of drug loading that was described as a “falling-off-the-cliff” effect, while the drug loading of HPMCAS ASDs had a smaller but still negative effect on the dissolution performance [10]. These results agree with other studies suggesting that HPMCAS can increase the dissolution and stabilize amorphous nanodroplets to a greater extent than other polymers, such as PVPVA, at higher drug loadings, as well as act as a precipitation inhibitor in vivo [12].

In addition to performance differences, the properties of each polymer limit the choice of ASD formulation technology and processing parameters that can be used [13]. For example, with solvent-based methods (e.g., spray-drying and solvent evaporation), the solvent system must be able to dissolve both the polymer and the API at sufficiently high concentrations for the economical manufacturing of ASDs [14]. On the other hand, while thermal methods such as hot melt extrusion do not require a common solvent, processing temperatures are limited to an extrudable range by the complex viscosity of the API–polymer system [15]. One potential advantage of solvent methods such as spray drying is the ability to create particles with a small particle size that can exhibit high dissolution rates [16]. The high-energy fusion technology KinetiSol compounding uses high-speed mixing without external heating to disperse and dissolve the API within a carrier (often polymeric) to form an ASD. KinetiSol compounding has a distinct advantage over both hot melt extrusion and spray-drying in terms of polymer choice in that it is neither limited by the complex viscosity of a polymer nor by its solubility in organic or aqueous solvents [17]. The use of KinetiSol compounding to formulate ASDs was recently reviewed in [18,19].

ASDs that contain more than one polymer as the carrier (i.e., multiple-polymer ASDs) are an interesting choice for overcoming the limitations of a single polymer. One way that multiple-polymer ASDs are formulated is by first adding APIs and polymers together, followed by physically mixing these components. This step is then followed by processing (e.g., solvent, thermal, or KinetiSol) to form an ASD. We refer to this method of making ASDs as the *polymer blending method* and to multiple-polymer ASDs made using this method as *polymer blends*. Polymer blends have been studied in ASDs for their ability to affect physical stability [7,20], as well as dissolution performance [21]. An important consideration for these polymer blend ASDs is whether each polymer is miscible or immiscible with the other polymer(s). It is often preferable for ASDs to be a single phase, where all components are miscible with the others at the desired ratio. Marks et al. examined the miscibility of multiple polymers, including HPMCAS, HPMC, PVP, and Eudragit 100 (a methacrylate copolymer), when prepared by spray-drying [22]. They determined that the cellulosic polymers (HPMC and HPMCAS) were miscible with PVP at all ratios but that at some ratios these two polymers were not miscible with Eudragit 100. The authors suggest that the ASD performance can be modulated using polymer blends containing hydrophobic and hydrophilic polymers. Despite the potential benefits of polymer blend ASDs, their use is comparatively limited in the literature because of the increased complexity when using polymer blends in ASDs compared to using a single polymer.

Yang et al. reported on the use of a ternary polymer blend of felodipine-Eudragit E PO-PVPVA ASD formulated using the polymer blending method by hot melt extrusion, which highlights the complicated nature of these polymer blend systems [23]. The two polymers chosen were immiscible with each other at most ratios following extrusion, as evidenced by the presence of two glass transition temperatures (Tg). Because of this immiscibility, when felodipine was extruded as a polymer blend combination containing equal parts by weight of PVPVA and E PO, the API was partitioned within each phase-separated polymer in unequal concentrations, where felodipine was found to be more miscible in the PVPVA. For the resulting ASDs, it was found that the hygroscopicity of the extrudate was a function of the polymer ratio, where PVPVA was more hygroscopic and E PO was less. Interestingly, an accelerated stability study found that at the highest drug loading (70% *w*/*w*), the polymer blend extrudate was amorphous by Fourier transform infrared (FTIR) spectroscopy and dynamic scanning calorimetry (DSC), while each extrudate with either E PO or PVPVA had evidence of crystalline API. In addition, at the lowest drug loading of 10%, the polymer blend ASD outperformed each single-polymer ASD in dissolution, although this benefit was not present at higher drug loadings. By combining an API with a polymer with a lower hygroscopicity (E PO) and one with a high miscibility (PVPVA), each polymer’s benefits could overcome the other’s limitations. Another work evaluated how the stability of ASDs comprising either naproxen or acetaminophen was affected by using different ratios of HPMCAS and either PVP or PVPVA in a polymer blend [7]. In this case, the formulations containing only HPMCAS had the worst stabilities at accelerated conditions due to the low miscibility of each API in this polymer. In contrast, the increased miscibility of the API in PVP and PVPVA outweighed their increased hygroscopicity, where ASDs containing 50–50 *w*/*w* PVP and HPMCAS as a polymer blend had an increased stability relative to the HPMCAS-only ASD, while the PVP/PVPVA-only ASDs had the highest stability. Here, any potential benefit in supersaturation or precipitation inhibition from the inclusion of HPMCAS into a polymer blend would need to be balanced against the decreased miscibility and physical stability from the inclusion of this polymer. Other work has examined the combination of two binary ASDs (each with posaconazole and either PVPVA or Eudragit RS PO) and reported a benefit in the dissolution performance due to the differences in the polymer solubility reducing the tendency of the API to recrystallize [24]. In contrast, it has been shown for other systems that polymer–polymer interactions can reduce the dissolution performance of ternary polymer blend ASDs if these interactions are too strong [25]. Despite this work in understanding the intricacies of polymer blend ASDs, these varied results reveal a large need for further investigation into the mechanisms for these altered ASD performances.

In this study, ASDs made from one or both (i.e., single- or multiple-polymer system) PVP and HPMCAS were evaluated for changes in processability and dissolution performance. Ivacaftor (IVA), which is used for the treatment of cystic fibrosis, was studied because it exhibits a low crystalline solubility (<0.05 µg/mL) and amorphous solubility (5–6 µg/mL) when un-ionized [11,26]. The marketed product, Kalydeco^®^, is reportedly formulated as an 80% drug load spray-dried dispersion (SDD) with the polymeric carrier HPMCAS to increase bioavailability [27]. Ivacaftor has previously been processed into an ASD by spray-drying, likely due to the fact of its high melting temperature of ~317 °C (Supplementary Materials Figure S1). KinetiSol compounding was chosen for this API because of its high shear mixing which has been shown to substantially reduce the necessary processing temperatures for high-melt APIs [28].

While the studies reported here have evaluated multiple-polymer systems made using the polymer blending method, scarce research has examined the effect of processing the polymers before addition to a physical blend in a subsequent ASD formulation. This study compared two ways to prepare an ASD by KinetiSol compounding: polymer blending method, as described above, and *polymer alloy method*. In contrast to the polymer blending method, the polymer alloy method begins with the physical mixing of only the polymers (without an API) that are subsequently processed by KinetiSol compounding to form a single-phase *polymer alloy*. This polymer alloy is milled into a suitable particle size distribution to form a powder and then mixed with API, whereupon this combination is processed into a polymer alloy–API ASD by KinetiSol compounding. Thus, the net effect of the polymer alloy process is to process the polymers twice by KinetiSol compounding, first, with only the polymer(s) and, second, with the created polymer alloy and the added API. The term polymer alloy has been defined as a “polymer mixture where one polymer phase is dispersed in another polymer phase” [29], and this term is used in this study to differentiate between an ASD made using the polymer blend method and an ASD made using the polymer alloy method. We examined and compared the effects of using PVP and HPMCAS (in equal parts by weight), formed into an IVA ASD by either the polymer blending method or the polymer alloy method, on the formulation of ASDs by KinetiSol compounding in terms of the drug loading, dissolution performance, and physical stability. The polymers and the ASDs were evaluated by solid-state characterization to understand changes in polymer–polymer and polymer–API interactions. In addition, we examined the effects of the particle size of KinetiSol-processed polymers on the subsequent processability of high drug-load ASDs with the KinetiSol processing technology. We hypothesized that using the polymer alloy method to prepare ASDs will provide the ability to tailor polymer properties of the polymer alloy to maximize drug loading, dissolution performance, and stability based on the needs of the particular ASD.

## 2. Materials and Methods

### 2.1. Materials

Ivacaftor was acquired from Nexconn Pharmatechs Ltd. (Shenzhen, China). Shin-Etsu Aqoat^®^ (Hypromellose Acetate Succinate) LMP were donated by Shin-Etsu, while Kollidon K30 (PVPK30) was donated by BASF Pharma. Biorelevant dissolution powder (FaSSIF/FeSSIF/FaSSGF) was obtained from Biorelevant Ltd. (London, UK). Sodium lauryl sulfate (SLS), NaCl, Na_2_SO_4_, microcrystalline cellulose, and croscarmellose were purchased from Fischer Scientific. Otherwise, ACS or equivalent grade chemicals were used. All compositions are weight by weight (*w*/*w*), unless otherwise specified.

### 2.2. KinetiSol Compounding

The polymer blend was prepared by combining equal parts by weight of PVPK30 and HPMCAS LMP. The mixture for the polymer alloy was prepared using a combination of 49.5–49.5–1 *w*/*w* of HPMCAS-PVPK30-SLS with manual mixing for 5 min. SLS was included as a surfactant in the polymer alloy, as well as in each ASD, to match the SLS present in the marketed product spray-dried dispersion. This mixture was then added into the KinetiSol formulator, where it underwent mixing at 1000 RPM for 10 s, followed by processing at 6250 RPM until ejected at 120 °C, which was reached by the viscous heating from the mixing. The ejected material was milled using an IKA Tube Mill (IKA-Werke, Stuafen, Germany) and sieved to <250 µm. The total milling time for any material was less than 5 min. Milling was performed in 30 s intervals that consisted of two 15 s pulses, where the milling speed increased from 0 RPM to 20,000 RPM. This ejected and sieved material was then used for all polymer alloy processing.

The physical mixtures of IVA and one of HPMCAS, PVPK30, polymer blend, or polymer alloy were prepared at a 20%, 40%, and 50% drug loading *w*/*w* with SLS added to reach a final weight percent of 0.5%. The compositions of each formulation are summarized in Table 1. These physical mixtures were processed by KinetiSol, as described above, with a final processing speed of 6250 RPM and an ejection temperature of 120 °C. All successfully ejected material was milled and sieved to <250 µm, and the unsieved material was re-milled until the sample could be sieved completely. The ejections of powder were collected without subsequent milling or sieving. The physical mixtures were prepared immediately before processing, and runs were conducted under ambient conditions and room air.

Cryomilled polymer alloy was prepared similarly, except that after the initial tube-milling to reduce the particle size, cryo-milling was conducted using an SPEX 6870 Freezer/Mill (SPEX SamplePrep, Metuchen, NJ, USA) cooled by liquid nitrogen. Cryomilling was conducted for 5 cycles of 5 min at 10 cps with 1 min cooling periods between each cycle.

### 2.3. Dynamic Vapor Sorption (DVS)

The hygroscopicity of pure HPMCAS, pure PVPK30, the polymer blend, and the polymer alloy were measured using a DVS Resolution (Surface Measurements, Allentown, PA, USA) gravimetric sorption system. Surface Measurements^®^ DVS control software was utilized for the analysis and experimental setup. The temperature for all experiments was 25 °C, and the carrier gas was nitrogen. The samples pans were tared and between 5 and 10 mg of each polymer or polymer mixture were dried at 0% RH until the change in the weight of the sample pan (i.e., dm/dt) was less than 0.002%/min. The water sorption of each sample was then measured at intervals of 10% RH from 0 to 90% RH, where the step-change occurred when dm/dt was less than 0.002%/min.

### 2.4. High-Performance Liquid Chromatography (HPLC)

A Waters HPLC unit equipped with an ultraviolet (UV) detector set at 245 nM (Waters Corporation, Milford, MA, USA) was used to measure IVA concentrations. An XBridge C18 3.5 µm, 4.6 × 150 mm column was used. Mobile phase A was 0.01N ammonium acetate buffer (pH 7.0), which was filtered prior to use, and mobile phase B was HPLC-grade methanol. The IVA method for determining the purity and potency used the gradient method shown in Table 2. The dissolution measurements were conducted using a constant elution ratio of 20:80 (A:B) at 1.2 mL/min at room temperature. The injection volume was 35 μL.

### 2.5. Modulated Differential Scanning Calorimetry (mDSC)

The thermal properties of KinetiSol-processed material, as well as physical mixtures and neat chemicals, were analyzed by mDSC (DSC 2920, TA Instruments, New Castle, DE, USA) using TA Universal Analysis 2000 software for data processing. The samples were prepared in crimped standard aluminum pans containing approximately 10 mg of material. The measurements were conducted at a ramp heating rate of 5 °C/min and a modulation of 1 °C/min from 50 to 250 °C after a 5 min equilibration at 50 °C [3]. All studies were conducted under a dry nitrogen gas purge of 50 mL/min. The glass transition temperatures are reported as their midpoint value.

### 2.6. Powder X-ray Diffraction (PXRD)

The amorphicity or crystallinity of IVA in different samples was examined by PXRD on a Rigaku Miniflex 600 II unit (Rigaku Americas, The Woodlands, TX, USA). The machine utilized a Cu-Ka radiation source set at 15 mA and 40 kV. The measurements were conducted over a 5–35° 2-theta (2θ) range with a scan rate of 2°/min and a 0.02° step size [3]. All samples were in powder form and added to the sample holder immediately prior to measurement.

### 2.7. Fourier Transform Infra-Red (FTIR) Spectroscopy

A Nicolet iS50 spectrometer (Thermo Scientific, Waltham, MA, USA) with OMNIC analysis software was used to measure the samples. The samples were evaluated in powder form using attenuated reflectance spectroscopy. Each sample had 64 scans conducted at room temperature from 4000 cm^−1^ to 675 cm^−1^ with a resolution of 4 cm^−1^. Background scans were subtracted from each sample to reduce the influence of water and carbon dioxide on spectra.

### 2.8. Tableting

The ASD products were formulated into tablets to increase their dispersibility in biorelevant dissolution media. A tablet additive comprising 25% NaCl, 25% Na_2_SO_4_, 25% microcrystalline cellulose, 24% croscarmellose, and 1% sodium lauryl sulphate (SLS), all by weight, was prepared by mortar and pestle. The tablet composition was selected to maximize the dispersibility due to the concerns of the gelling of PVP-containing ASDs. The ASD was combined with this tablet additive at a 60:40 ratio and mixed thoroughly by mortar and pestle before tableting. Tableting was conducted using a manual BVA Hydraulics tableting press (Shinn Fu America, Kansas City, MO, USA) with an oblong die (6.3 mm by 19.0 mm) compressed at 2000 psi for 5 s.

### 2.9. Dissolution Study

A non-pH transition dissolution test was chosen to avoid changes in release that would only be caused by the solubility differences of HPMCAS. As IVA’s ionization state does not change in this range of physiological pH values (pKa of 9.4 and 11.6), a pH transition study was not expected to provide additional biorelevant dissolution information. Non-sink dissolution testing in biorelevant media was conducted on ASDs and physical mixtures in either neat powder or tablet form using a Hanson SR8PLUS dissolution apparatus (Hanson Research Co., Chatsworth, CA, USA). The temperature of the apparatus was held at 37.5 °C ± 1.0 °C with a constant paddle rotation rate set to 100 RPM for all tests. KinetiSol ASDs were milled and sieved to obtain <250 μm particle size for all samples. The theoretical maximum concentration of each vessel was set to 1000 µg/mL by the addition of the equivalent of 150 mg of IVA. For non-pH transition experiments, 150 mL of pH 6.5 fasted-state simulated intestinal fluid (FaSSIF) or solution containing phosphate buffer was added to 200 mL vessels and equilibrated for 1 h prior to sample addition. Samples were taken at 5, 10, 20, 30, 45, 60, 120, 240, and 360 min for non-pH transition tests and filtered using 0.45 μm polytetrafluoroethylene (PTFE) filters. An aliquot of 1000 μL of MeOH was used to dilute 250 μL of the filtrate for HPLC analysis.

### 2.10. Rhodos Particle Size Analysis

The particle size distribution of the tube and cryo-milled polymer alloy prepared by KinetiSol compounding, as well as spray-dried polymer alloy, was evaluated using Helos (Sympatec, Clausthal-Zellerfeld, Germany) laser diffraction with a RODOS dispersion of 3 bars. The motor speed was set to 50%, and vacuum was used. After dispersion, measurements were taken at 10 ms intervals. The measurements when the optical density was between 5 and 25% were averaged to determine the distribution of the particle sizes. Fraunhofer theory was used to solve the particle size distribution.

### 2.11. Solid-State Nuclear Magnetic Resonance (ssNMR)

A Bruker Avance III HD spectrometer (Bruker, Billerica, MA, USA) was used to perform the 13C ssNMR experiments. The samples were packed under ambient conditions into 4 mm rotors [3]. The spectra were obtained from 4096 scans with an 8 kHz spinning frequency. The external standard used was glycine, where its carbonyl carbon peak was set at 176.02 ppm and used as calibration for all 13C chemical shifts. The data analysis was conducted using Mnova version 14.3.1 (Mestrelab Research, Santiago de Compstela, Spain).

## 3. Results and Discussion

### 3.1. KinetiSol Compounding Creates a Single-Phase Polymer Alloy

An equal weight mixture of HPMCAS L grade and PVPK30 (with 1% *w*/*w* SLS as a surfactant) was selected as the mixture that would be processed by the polymer alloy method into a polymer alloy. These two polymers were chosen to include one comparatively hydrophilic polymer (PVPK30) and one hydrophobic polymer (HPMCAS) that were miscible at the selected ratio. The SLS was included to match the IVA ASDs described in the Kalydeco^®^ patent [27]. While previous work has established that a mixture of these two polymers can form a single phase via spray-drying, the creation of a single-phase combination of this mixture has not been conducted using KinetiSol compounding [22]. This polymer mixture was processed in a KinetiSol formulator at 6250 RPM and ejected at 120 °C as a molten mass to confirm its ability to create a single-phase system of this polymer combination. The ejected polymer alloy was collected, milled, and used to create IVA polymer alloy KSDs.

**Figure 1:**
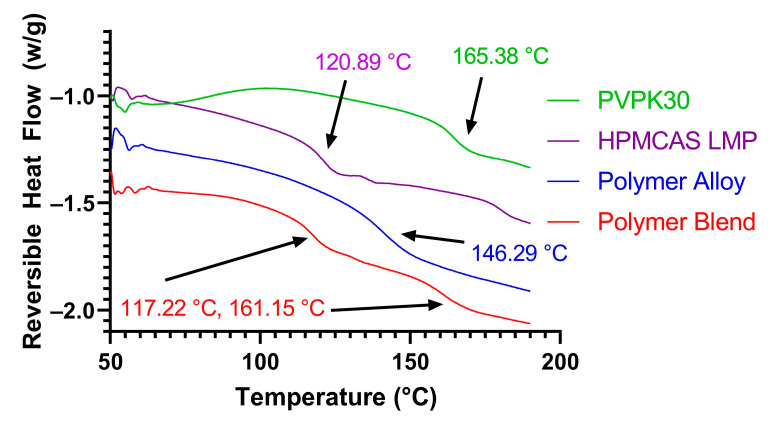
mDSC of pure PVPK30, pure HPMCAS LMP, polymer blend, and polymer alloy. The Tgs of each composition are indicated by the arrows. A decrease in the reversible heat flow indicates an endothermic event.

The two neat polymers, an equal weight physical mixture of HPMCAS and PVPK30 (i.e., polymer blend), and the polymer alloy were analyzed by mDSC to determine the glass transition temperature(s) present (Figure 1). The Tg of each of the pure polymers was in agreement with the literature at approximately 120 °C (120.89 °C measured) for HPMCAS L and 163 °C (165.38 °C measured) for PVPK30. In addition, the polymer blend had two Tgs near the temperatures of each individual polymer, as would be expected from an unprocessed combination. The processed polymer alloy was determined to be a single-phase combination of the two, as only a single Tg at 146.29 °C was measured by mDSC. The measured value was in good agreement with the Gordon–Taylor expected Tg of 138.83 °C for this combination, although there was a positive deviation of ~7 °C. Positive deviations from the Gordon–Taylor value can be due to the presence of stronger hetero-polymer interactions (e.g., PVPK30-HPMCAS) compared to homo–polymer interactions (e.g., PVPK30-PVPK30 or HPMCAS-HPMCAS) [30]. As HPMCAS contains multiple hydrogen-bonding donor moieties and PVPK30 contains a carbonyl group that can act as a hydrogen bond acceptor, it is likely that this interaction is contributing to the increased Tg. The intermolecular interactions of the polymer alloy and constituent polymers are examined in depth in Section 3.5.

**Figure 2:**
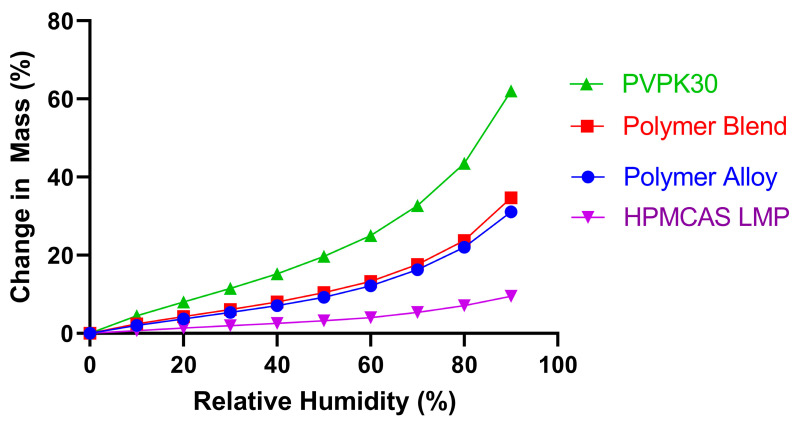
Change in mass of the pure polymers, as well as polymer blend and polymer alloy, as measured by dynamic vapor sorption (DVS) as a function of the relative humidity.

The hygroscopicity of the polymers was also evaluated. The results of the dynamic vapor sorption (DVS) measurements for the pure PVPK30, pure HPMCAS, unprocessed polymer blend (equal weight HPMCAS and PVPK30), and polymer alloy are depicted in Figure 2. As expected, PVPK30 had the greatest water uptake at all relative humidities (RH) tested; at 50% RH, the change in mass from the baseline had already reached approximately 20%. In contrast, HPMCAS L had the lowest measured hygroscopicity and only absorbed slightly over 3% water at 50% RH. The unprocessed polymer blend change in mass was similar to the average expected from an equal part mixture of the two neat polymers, and the processed polymer alloy values were modestly lower than the polymer blend. These results show that the polymer alloy method of processing does not substantially change the hygroscopicity of the polymer alloy compared to a compositionally similar polymer blend. It also confirms that the overall hygroscopicity of the system can be modified by the relative component mass of the constituent polymers. The ability to modify the hygroscopicity of an ASD system has important implications if the presence of water causes a reduction in its physical or chemical stability. In addition, it has been suggested that the hydrophilicity of a polymer (as measured by its water sorption profile) affects both the dissolution performance and the maximum drug loading of an ASD [31].

### 3.2. The Polymer Alloy Method Increases the Maximum Drug Loading in KinetiSol Ivacaftor ASDs

To understand the effect of polymer choice on the maximum achievable drug loading of IVA amorphous solid dispersions, compositions containing 20–50% *w*/*w* IVA, and one of HPMCAS, PVPK30, polymer blend, or polymer alloy were processed using KinetiSol compounding. Each processed sample was examined by PXRD and mDSC to determine if residual crystalline IVA was present and by HPLC to evaluate for purity and potency. The processing conditions, including ejection temperature, blade rotation speed, mass processed, and maximum processing time, were held constant. These conditions were intentionally not optimized for each formulation to better understand the differences in processability due to the formulation compositions. The ejection temperature was set to 120 °C, nearly 200 °C below the measured melting point of IVA (317.2 °C, Supplementary Materials Figure S1).

**Figure 3:**
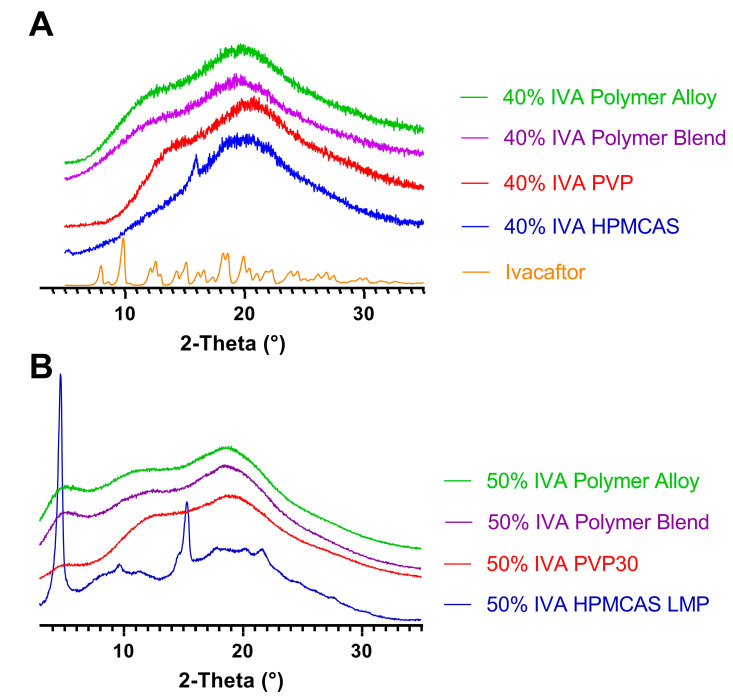
PXRD of (**A**) 40% (*w*/*w*) IVA ASDs and (**B**) 50% IVA ASDs formulated by KinetiSol, as well as pure IVA. The intensity units are arbitrary, and the data are superimposed for visual clarity.

At the 20% IVA loading, the formulations containing the individual polymers, the polymer blend, and the polymer alloy were all amorphous by mDSC and PXRD. In contrast, at the 40% loading, the 40% IVA HPMCAS exhibited two small, new Braggs peaks that did not correspond with the initial IVA polymorph, while the other formulations were all amorphous by PXRD (Figure 3A). It is possible that the new Braggs peak in the 40% IVA HPMCAS ASD was due to the temperature-dependent recrystallization event.

**Figure 4:**
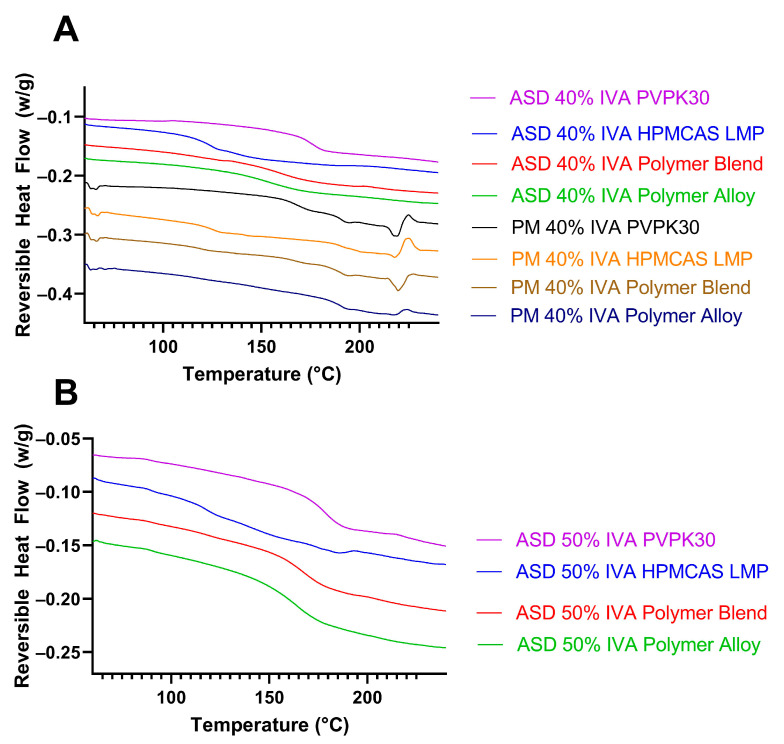
mDSC of (**A**) physical mixtures (PM) and KinetiSol-processed 40% IVA ASDs and (**B**) KinetiSol-processed 50% IVA ASDs. An endothermic event between 185 and 190 °C or 215 and 220 °C is indicative of the presence of crystalline IVA. The thermograms are superimposed for visual clarity.

The interpretation of mDSC for IVA is complicated due to the multiple thermal events that occur when pure IVA is heated to its high melting point. Despite this complication, the mDSC of the physical mixtures of 40% IVA with each polymer or polymer combination revealed two endothermic events associated with crystalline IVA: one between 195 and 200 °C and the other between 215 and 220 °C (Figure 4A). Because of the degradation of polymer above ~250 °C, the melting point of IVA was not directly measurable in these formulations. In agreement with PXRD results, the three formulations containing PVP (PVP, polymer blend, and polymer alloy) had a single Tg, suggesting that the ASD was a single phase without measurable crystalline IVA. In contrast, the mDSC of the 40% IVA HPMCAS ASD disagreed with PXRD, which suggests a small quantity of crystalline material, by showing no thermal events associated with crystalline IVA. The Tg of each respective amorphous 40% IVA system was 155.19 °C for the polymer alloy, 160.82 °C for the polymer blend, and 175.01 °C for the PVPK30. The 40% IVA HPMCAS system had a distinct Tg at 124.16 °C in addition to a smaller endothermic event at 172.16 °C, suggestive of partially phase-separated amorphous IVA, whose neat amorphous Tg has been reported as 175 °C [32]. It was determined that the examined systems, except for 40% IVA HPMCAS, were single-phase, amorphous systems by PXRD and mDSC. The lack of a distinct melting event in the 40% IVA HPMCAS was not unexpected, as previous studies have shown that it can be difficult to detect the presence of relatively small quantities of crystalline material by DSC or mDSC if the material dissolves in the chosen polymer instead of melting [3,33,34].

**Figure 5:**
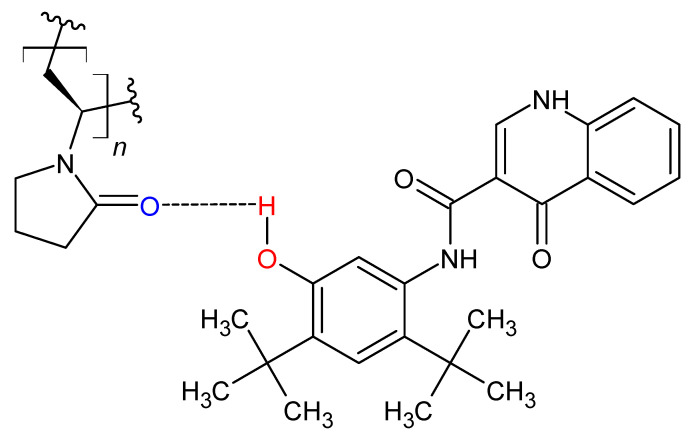
Molecular structure of PVPK30 (left) and IVA (right) with proposed hydrogen bonding between IVA phenol and PVPK30 carbonyl moieties.

Next, the formulations containing 50% IVA were also processed using KinetiSol and examined by PXRD (Figure 3B) and mDSC (Figure 4B). At this drug loading and chosen set of processing parameters, it was found that while the HPMCAS- and polymer-alloy-containing compositions could be processed and ejected as continuous molten masses (i.e., the normal result of KinetiSol compounding), the PVP and polymer blend formulations were unable to be processed and instead ejected as powder. Surprisingly, PXRD showed that the PVPK30, polymer blend, and polymer alloy were all amorphous at the 50% drug loading, despite the incomplete processing of the PVP and polymer blend, at these fixed processing conditions. These results suggest that IVA has a high miscibility with PVPK30 to be converted amorphous without complete processing; this high miscibility could be caused by hydrogen bonding between the phenol group of the IVA and the carbonyl group of PVPK30 (Figure 5), as hydrogen bonding between an API and a polymer is often associated with miscibility [35]. The 50% IVA HPMCAS processed material was crystalline by PXRD, and the new Braggs peaks had a greater intensity compared to the 40% IVA HPMCAS KinetiSol compounded material, supporting that the increased drug loading promoted recrystallization. The mDSC results were in agreement with the PXRD, where the PVP, polymer blend, and polymer alloy 50% IVA ASDs each had a single Tg of 179.54 °C, 167.53 °C, and 164.03 °C, respectively. For each of these ASD formulations, there was no endotherm associated with crystalline IVA. The 50% IVA HPMCAS material had no clear single Tg and a small endothermic event at ~185 °C that was potentially caused by the new crystalline IVA polymorph dissolving in the HPMCAS, indicating it was not a single-phase and amorphous ASD.

Based on these results and under these processing conditions, the only fully amorphous and successfully processed 50% IVA ASD was the one containing the polymer alloy and processed by the polymer alloy method. While HPMCAS demonstrated robust processibility by achieving a molten ejection at all drug loadings tested, it appears to undergo a drug-load-dependent recrystallization of IVA. In contrast, PVPK30 exhibited a high miscibility with IVA during the KinetiSol compounding but was incompletely processed at the fixed processing conditions. The combination of the PVPK30 and the HPMCAS into the polymer blend at a 50% IVA loading overcame the recrystallization seen with HPMCAS but lost its processing benefit, as it also ejected as an underprocessed powder. The difficulty in processing the PVPK30 and polymer blend under these conditions may have been due to the comparatively smaller particle size of the PVPK30, where up to 40% *w*/*w* of the PVPK30 particles can be under 50 µm [36]. In contrast, the HPMCAS exhibits larger particles with an average particle size of ~200 µm [37]. KinetiSol compounding relies on high-shear mixing to impart energy via viscous heating into the formulation [38]. For this reason, larger particles are better able to resist this mixing (and in doing so, become deformed and experience increased dispersive mixing), and because of this, energy is efficiently transferred to them via interparticle friction that imparts heat. In contrast, smaller particles are fluidized by the rotating blades and avoid viscous heating, making processing more difficult. The effect of the polymer particle size on the processability of these formulations is examined in Section 3.3. By using the polymer alloy method to process the polymer blend into the polymer alloy, it was possible to create the only 50% IVA ASD that was both amorphous and completely processed with a high yield. A 60% IVA polymer alloy formulation was also tested but was ejected as powder, indicating that there were still limitations to using the polymer alloy to increase processable drug loads. It is important to again note that the processing parameters were held constant for all compositions to better understand how the characteristics of the compositions affected the processability and that optimization for each composition was not undertaken.

The results of this study show the ability of the KinetiSol compounding technology to enhance mixing during processing to allow compositions to be rendered amorphous, even above the miscibility in the polymer (i.e., 40% IVA in HPMCAS). Because of this enhanced ability, as well as if the molecule has a strong tendency to recrystallize, this recrystallization during KinetiSol compounding can occur, as seen in the HPMCAS ASDs. Despite this possibility, the combination of PVPK30 and HPMCAS and pre-processing into the polymer alloy aided in overcoming this challenge. These results also suggest that the design and creation of polymer alloys may be a useful tool in ASD formulation in addition to decisions concerning the polymer choice and the ratio of polymers within a blend.

### 3.3. Increased Particle Size of Polymers during KinetiSol Compounding Increases Maximum Drug Loading

It was hypothesized that the creation of the polymer alloy affected the processability of these ASDs due to the change in particle size. It has been shown previously that the particle size (particularly of the smallest size fraction) can alter the particle cohesion and flowability, where larger particles are less cohesive with better flowability [39]. It was expected that a reduction in the particle size would, in general, reduce the processability by KinetiSol due to the lowered flowability. To test this hypothesis concerning the effect of the particle size of the polymer alloy on the KinetiSol processability, two comparators with differing particle sizes were prepared. Polymer alloy was milled either using tube-milling (method used to mill polymer alloy for all other experiments) or by cryo-milling. The particle sizes of the tube-milled polymer alloy were greater than those of the cryo-milled polymer alloy. The decreased particle size of the cryo-milled particles was due, at least in part, to the increased milling time of 25 min compared to less than 5 min for the tube-milled. These particle sizes are summarized in Table 3.

KinetiSol compounding using each of these two milled polymer alloys was attempted at the selected processing parameters described in Section 3.2 to identify differences from particle size changes. As reported in Section 3.2, the polymer alloy (tube-milled) could be processed and ejected as a single phase with a maximum drug loading of 50% IVA but was unprocessable at 60% IVA. The smaller cryo-milled polymer alloy was also processable with a 50% IVA loading, but the total processing time was increased from ~10 s at elevated RPMs to ~34 s prior to ejection. The increased processing time is indicative of the relative difficulty in processing and reaching the chosen ejection temperature. These results suggest that selecting a larger particle size range can improve the processability of formulations using KinetiSol compounding. These results are in agreement with a recent study that describes a modest increase in the processability of a novel copovidone polymer (Plasdone™ S630 Ultra) during the hot melt extrusion of quetiapine ASDs, which was attributed to improved flowability compared to another copovidone [40].

To further probe the effect of particle sizing on processability, pure PVPK30 was processed by KinetiSol compounding using the polymer alloy method, and the ejected material was milled and sieved to obtain particles between 500 and 1200 µm. These particles of the polymer-alloy-processed PVPK30 were substantially larger than the those from the unprocessed PVPK30, which were in the approximate range of 50–250 µm [36]. This pre-processed PVPK30 was then mixed with IVA to create mixtures of up to 70% *w*/*w* IVA and processed by KinetiSol. While the unprocessed, smaller-particle-sized PVPK30 was processable at these conditions only up to 40% IVA, the larger-particle-sized PVPK30 prepared using the polymer alloy method could be processed with a maximum of 70% IVA. The 70% IVA PVPK30 was determined to be amorphous by PXRD and mDSC (Supplementary Materials Figure S2) and had no process-related impurities as determined by HPLC. These results support both that IVA is highly miscible with PVPK30 (as evidenced by the high achievable drug load) and that a larger particle size created by polymer alloy processing can aid substantially in the processability of high drug-load ASDs during KinetiSol. Future work to examine the potential for particle size modification to alter processability by KinetiSol compounding would aid in the formulation of ASDs with increased drug loadings.

### 3.4. High Drug-Load KinetiSol ASDs Achieve High and Sustained Supersaturation

**Figure 6:**
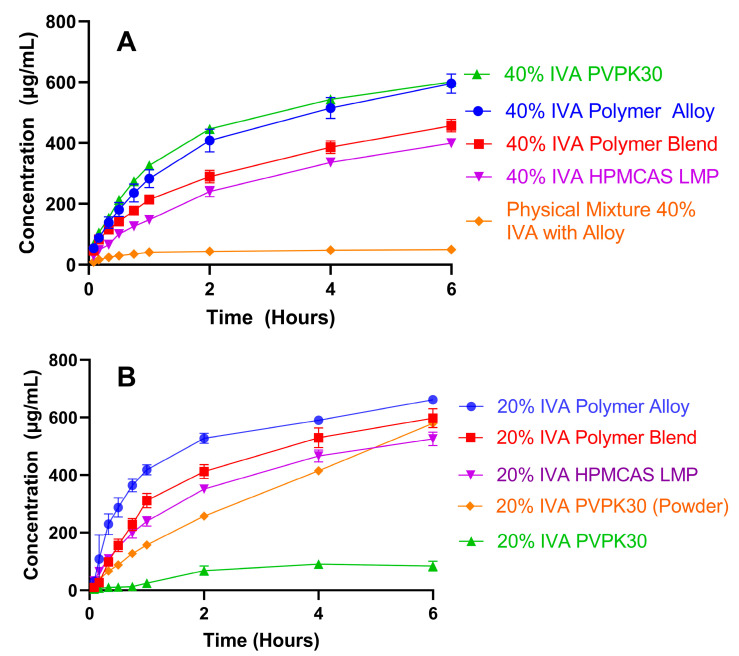
Dissolution concentrations of IVA in a FaSSIF medium (pH 6.5, *n* = 3): (**A**) tablets containing 40% IVA ASDs and physical mixture; (**B**) tablets containing 20% IVA ASDs in addition to 20% IVA PVP ASD as a powder. The theoretical max. concentration was 1000 µg/mL, and the crystalline solubility of IVA was ~50 µg/mL in FaSSIF. Note that the 40% IVA HPMCAS ASD had residual crystalline IVA.

Dissolution studies in pH 6.5 FaSSIF (37.5 °C ± 1.0 °C) were conducted to understand how the polymer choice and processing method (i.e., polymer blending vs. polymer alloy) affected the rate and extent of IVA dissolution in intestinal media. A FaSSIF medium was chosen as the dissolution medium because of its intermediate ability to solubilize IVA during dissolution. Previous work has shown that IVA ASD dissolution in buffered pH 7.5 media is low at drug loadings above ~10–15% in PVPVA- and HPMCAS-containing ASDs, while a FeSSIF medium aids in dissolution to such an extent that the differentiation among formulations could be difficult [11,38]. Both the 40% and 20% IVA ASDs were evaluated, as drug loading has been shown to alter the dissolution of IVA PVPVA and HPMCAS ASDs [11]. Due to the initial dispersibility challenges with free ASD powders, a uniform tablet composition was created to standardize the dispersibility at a 40% IVA ASD drug loading. Tablet compositions are summarized in Supplementary Materials Table S1. The results of these studies are shown in Figure 6A. Overall, the rate and extent of IVA dissolution from the 40% IVA ASDs were ordered (greatest to least) as PVPK30 ~ polymer alloy > polymer blend > HPMCAS. In this case, the high miscibility with PVPK30, as evidenced from the greater maximum drug loading (70% IVA PVPK30 amorphous vs. 40% IVA HPMCAS, which was unable to prevent recrystallization during processing) appears to have provided a substantial dissolution benefit to the ASD containing only PVPK30. In comparison to the PVPK30 ASD, the polymer alloy had a slightly lower dissolution rate for the first hour but reached the same concentration at 6 h, indicating that the pre-processed alloy aids in the dissolution to nearly the same extent as the PVPK30 ASD. This result is surprising due to the comparatively lower dissolution of the 40% IVA polymer blend that behaves more similarly to the 40% IVA HPMCAS KSD, which had the lowest dissolution extent. In this case, the polymer alloy had a dissolution performance greater than the average of the two neat polymer formulations, while the polymer blend had a lower dissolution performance. It should also be noted that at 40% IVA, the HPMCAS ASD had a residual crystallinity that would be expected to worsen the dissolution performance, so the similar performance of the polymer blend (no measured residual crystallinity) is unexpected.

To ensure that the reduced dissolution from the 40% IVA HPMCAS ASD was not due to the measurable residual crystalline material, the dissolution of tablets containing the 20% IVA ASDs of each polymer and polymer combination was also tested (Figure 6B). While the previously seen dissolution trend of alloy > blend > HPMCAS held for these 20% drug load formulations, the PVPK30 formulation unexpectedly underperformed and reached only ~100 mcg/mL concentrations (~10% drug release). Visual examination of the tablets during dissolution revealed that while the other formulations disintegrated relatively rapidly (i.e., <20 min), the 20% IVA PVPK30 tablet did not disintegrate over the 6 h experiment. An additional disintegration test under identical USP II dissolution conditions (media, paddle speed, and temperature) revealed that this tablet failed to disintegrate even after 30 h. While PVP is often a dissolution aid when used to create ASDs [8,35], PVP can also be used as a binder for sustained-release formulations to slow disintegration and dissolution. One study examined the effect of the composition on the rate of ibuprofen drug release from a hydroxypropyl methyl cellulose (HPMC)-based sustained-release tablet [41]. It was found that increasing the PVP from <1% to 5% *w*/*w* resulted in the drug release decreasing from 100% to less than 40% over 10 h. Another study examined the dissolution of pressed tablets containing ASDs of itraconazole and PVPK30 at a 20:80 drug:polymer ratio prepared by hot melt extrusion and KinetiSol compounding [42]. The dissolution of these pressed tablets revealed substantially slower drug release in acidic media than by equivalent ASD granules, where the granules achieved ~50% release by 9 h, while the compressed tablets reached only ~15% release by 24 h. This change in dissolution was hypothesized to be due to the ingress of water into the tablet that caused precipitation of the drug within the tablet [42].

These results show how modest changes in formulation composition and dosage form can alter drug release. In comparison to the tablet containing 40% IVA ASD, which had an overall tablet PVP weight percent of 36%, the tablet containing the 20% IVA ASD was 48% PVP by weight. It appears that while PVP aids in the dissolution of IVA ASDs at lower polymer percent masses, when PVP comprises a sufficiently high percentage of the overall oral formulation (in this case, a compressed tablet), a reduced disintegration due to the fact of gelling reverses this trend. In support of this, the dissolution performance of the 20% IVA PVPK30 milled ASD powder (particle size < 250 µm) was also evaluated and showed acceptable dissolution at 6 h (Figure 6B), although the dissolution performance was lower than would be expected if delivered as an optimized tablet, as discussed at the beginning of this section. Overall, the dissolution results of both the 40% and the 20% IVA ASDs were in agreement that the polymer alloy ASDs had an increased dissolution rate and extent compared to what would be expected using a simple average of the corresponding PVPK30 and HPMCAS ASDs. In contrast, the polymer blend had a dissolution rate near or below this expected average.

The increased dissolution extent of the polymer alloy suggests a synergistic mechanism of release over that achieved by a simple average of the two single-polymer ASDs. Previously, Que et al. demonstrated that for ASDs containing PVPVA (a copolymer comprised of PVP and polyvinyl acetate), changes in the ability of an API to engage in intermolecular interactions (in this case, hydrogen or halogen bonding) with the polymer dramatically changed the dissolution performance at different drug loadings [43]. Additionally, because IVA has a phenolic group that can theoretically engage in hydrogen bonding with the amide carbonyl group of PVPK30 (Figure 5), it was hypothesized that the pre-processing of PVPK30 with the HPMCAS into polymer alloy may have altered the ability of the PVPK30 to hydrogen bond with IVA during the subsequent KinetiSol compounding.

### 3.5. Modification of Hydrogen Bonding in Polymer Alloy Explains Dissolution Benefit

**Figure 7:**
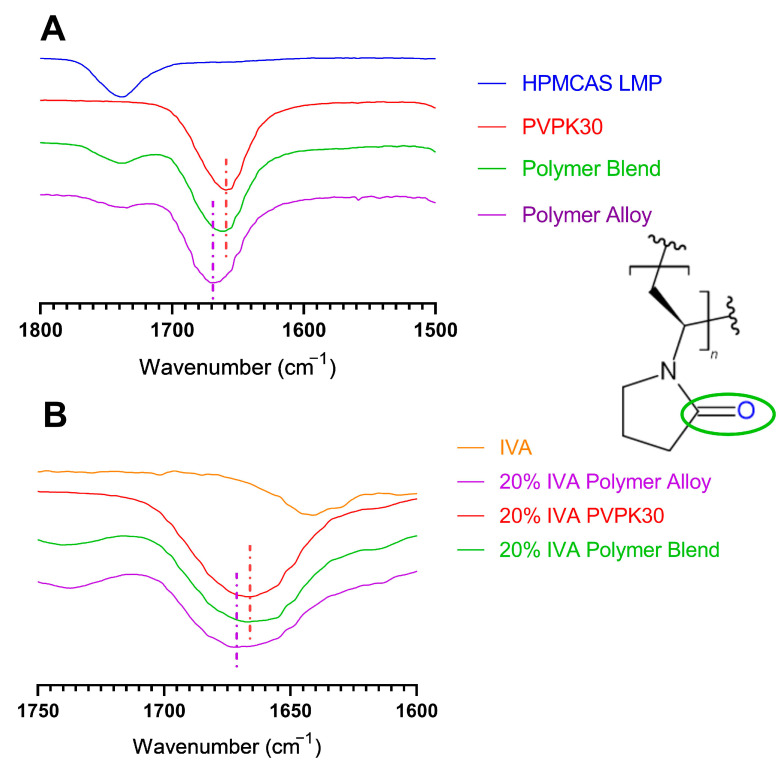
FTIR centered on PVPK30 carbonyl moiety: (**A**) pure PVPK30, HPMCAS LMP, polymer blend, and polymer alloy; (**B**) pure ivacaftor (IVA) and 20% IVA ASDs with PVPK30, polymer blend, or polymer alloy. The PVPK30 structure and carbonyl moiety (green oval) are shown to the right of the graphs. The colored, dashed lines are matched to their respective spectra and indicate the wavenumber at which a peak signal occurs. Spectra are superimposed for visual clarity.

Studies using FTIR and ssNMR were used to evaluate the intermolecular interactions of polymers and IVA to understand changes in the dissolution performance of these ASDs. FTIR was conducted on each of the neat polymers (PVPK30 and HPMCAS), as well as the polymer blend (physical mixture), and the milled polymer alloy. The polymer alloy showed no new peaks supporting that degradation of the PVPK30 pyrrolidone ring did not occur during processing. On the other hand, FTIR revealed changes in the chemical environment of the carbonyl carbon present in PVPK30, whether alone or as part of either the polymer blend or the polymer alloy (Figure 7A). The carbonyl carbon (as part of the amide group) in neat PVPK30 had a wavenumber of 1659.28 cm^−1^, which correlates to the C=O stretch of an amide group [44].

It has previously been reported that the carbonyl group in PVP engages preferentially in hydrogen bonding over the nitrogen of the pyrrole ring due to the fact of the significant steric hindrance of the nitrogen, so this wavenumber was singled out for examination [45]. Additionally, as PVPK30 is a highly hygroscopic material, it would be expected that there would be water present to hydrogen bond with this carbonyl unless specifically dried. It has been previously reported that the amide carbonyl of pure PVP under dry conditions has a maximal signal at 1682 cm^−1^ [44]. This signal shifts to a lower wavelength when engaged in hydrogen bonding (1655 cm^−1^ in the case of curcumin-PVP ASD) [44]. Another study showed a similar increase in the wave number for “completely dried” PVPK30 compared to one containing 2% water [8]. For this reason, the 1659.28 cm^−1^ wavenumber measured in pure and undried PVP suggests the presence of hydrogen bonding with absorbed water. Because these experiments were intended to examine the conditions experienced by the polymers before and during processing that contributed to the changes in ASD formation, the polymers were allowed to rest at ambient temperatures and humidity for at least 24 h before measurement.

For the polymer blend, this C=O stretch was found at 1660.54 cm^−1^, which is similar to the 1659.28 cm^−1^ of pure PVPK30. In contrast, for the processed polymer alloy, the wavenumber was substantially higher at 1670.20 cm^−1^, suggesting a meaningful difference in the microenvironment of the PVPK30 in the alloy compared to the pure PVPK30 and the polymer blend. Based on the higher wavenumber of this PVPK30 in the polymer alloy, we hypothesize that absorbed water was displaced due to the thermal processing during the polymer alloy method. Once that water was displaced, the PVPK30 within the single-phase polymer alloy was then less able to engage in hydrogen bonding because of the presence of HPMCAS, a more hydrophobic polymer. The essentially identical signal from the pure PVPK30 and the polymer blend shows that thermal processing is a necessary step in this change. To test this hypothesis, each of the PVPK30-containing mixtures was dried in a vacuum oven for 24 h at 50 °C. It was found that after drying, the carbonyl carbon peak in the neat PVPK30 (1674.14 cm^−1^), polymer blend (1671.67 cm^−1^), and polymer alloy (1675.74 cm^−1^) all shifted to higher, and similar, wavenumbers, indicating the changes seen are likely due to the presence or absence of water.

The presence or absence of PVPK30–water hydrogen bonding was hypothesized to alter the ability of each system to hydrogen bond with IVA during processing. Because of this, the FTIR spectra of the 20% IVA ASDs of each polymer, polymer blend, and polymer alloy were collected to identify potential hydrogen bond changes (Figure 7B). IVA has a phenolic group that can act as a hydrogen bond donor to the carbonyl (hydrogen bond acceptor) in PVPK30, and changes in the wavenumber of the carbonyl signal or the phenolic carbon can reveal changes in the hydrogen bonding of the polymer and IVA. It was assumed that due to the 120 °C ejection temperature, which is above the boiling point of water, and the rapid mixing during the KinetiSol compounding, the existing hydrogen bonding with water would largely be disrupted during processing. The carbonyl stretch signals for the 20% IVA PVPK30 and polymer blend ASDs were nearly identical at 1666.71 cm^−1^ and 1667.21 cm^−1^, respectively, suggesting that the carbonyl carbon was engaged in similar intermolecular interactions for each formulation. In contrast, this signal for the 20% IVA polymer alloy was at a slightly higher wavenumber of 1671.57 cm^−1^. This increased wavenumber for the PVPK30 carbonyl carbon in the polymer alloy indicates a relative deshielding that is suggestive of reduced hydrogen bonding between the phenolic group of IVA and the polymer alloy. While FTIR also evaluated the 40% IVA ASDs, the presence of an IVA carbonyl peak at ~1640 cm^−1^ [46] made interpretation difficult due to the substantial overlap seen between this peak and the PVPK30 peak. Additionally, despite apparent changes in the peaks of PVPK30 in each composition, the FTIR of HPMCAS lacked obvious changes. This lack of changes could be caused by the presence of a variety of similar functional groups or by minimal hydrogen bonding between HPMCAS and the other components. Overall, while the FTIR spectra were suggestive of changes in the hydrogen bonding capacity of PVPK30, either alone or within the polymer alloy, it was decided that another technique was necessary for further elucidation.

**Figure 8:**
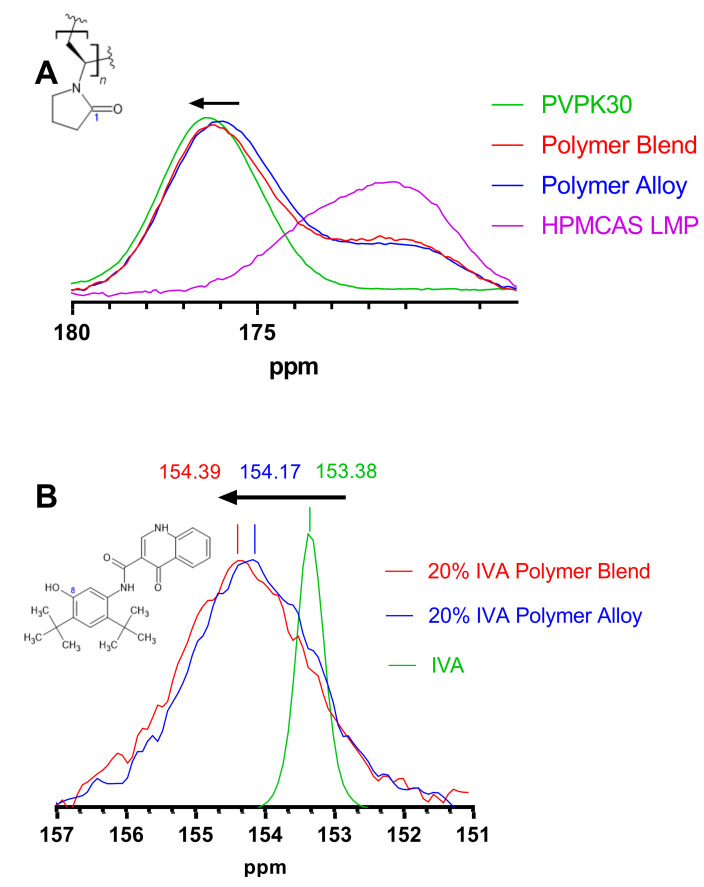
The 13C ssNMR of (**A**) pure PVPK30 and HPMCAS LMP, polymer blend, and polymer alloy and (**B**) 20% IVA polymer blend and polymer alloy ASDs and pure ivacaftor (IVA). (**A**) The peaks centered at 176 ppm are assigned to the carbonyl carbon of PVPK30 (labeled “1”); the chemical shift of (pure) PVPK30 is 176.33, polymer blend 176.11, and alloy 175.90 ppm. (**B**) The shown peak corresponds to the phenolic carbon of IVA (labeled “8”). The arrows show downfield shifting indicative of increased hydrogen bonding.

Solid-state nuclear magnetic resonance was conducted on each of the polymers and combinations, as well as on the 20% and 40% IVA ASDs with polymer alloy or polymer blend, to identify any changes due to the fact of hydrogen bonding. The carbonyl carbon of PVPK30 has previously been assigned near 176 ppm by 13C ssNMR [47]. In general, a downfield shift (i.e., increase in ppm) is indicative of the deshielding of a molecule, which is often caused by hydrogen bonding. Previous work has shown that when PVP and an API that can act as a hydrogen bond donor are formulated as an ASD, the PVP carbonyl carbon can experience a downfield shift [47]. The chemical shifts of the carbonyl carbon of PVPK30 and HPMCAS are shown in Figure 8A. In agreement with the FTIR results, the pure PVPK30 is engaged in hydrogen bonding (presumably with water) to the greatest extent with a peak at 176.33 ppm. The carbonyl carbon of the PVPK30 component of the polymer blend has a slightly upfield peak at 176.11 ppm, while within the polymer alloy its peak was 175.90 ppm, resulting in a change in ppm of 0.42 relative to pure PVPK30. Chemical shifts in ssNMR on the order of 0.2–0.5 ppm of this PVP carbonyl carbon have been attributed to changes in the hydrogen bonding as a function of the drug loading of rafoxanide-PVP ASDs [47]. In contrast, the PVP methylene backbone carbon has a maximum at 31.44 ppm for both the 20% IVA polymer blend and polymer alloy ASDs. Additionally, the quaternary carbons of the tert-butyl groups have maximums at 34.82 ppm and 34.85 ppm for the 20% IVA polymer blend and 20% IVA polymer alloy, respectively. The minimal changes in ppm (i.e., ≤0.03 ppm) for each of these carbon peaks are indicative of a lack of change in shielding; this lack of change is expected because these carbons would not be anticipated to engage in hydrogen bonding. These two peaks are shown in Supplementary Materials Figure S3. These results support that the polymer alloy PVPK30 carbonyl is engaged in hydrogen bonding to the least extent.

The chemical shift of the phenolic carbon of IVA was examined to determine if the change in the hydrogen bonding of PVPK30 would be correlated with hydrogen bonding within each ASD (Figure 8B). The phenolic carbon of crystalline IVA (153.38 ppm) undergoes a downfield shift in both of the 20% ASDs, showing that it likely engages in hydrogen bonding in both systems [48]. The peak broadening of the phenolic carbon signal is indicative of its conversion to an amorphous form, and this phenomenon has been reported previously [49]. While there appears to be hydrogen bonding in each system, as each peak is downfield compared to that of pure IVA, the phenolic carbon of the 20% IVA polymer blend is 0.22 ppm downfield of the 20% IVA polymer alloy. This suggests that the phenolic carbon is engaged in hydrogen bonding to a greater extent than in the 20% IVA polymer alloy. The 40% IVA ASDs were also evaluated for each of these changes, but overlapping peaks from IVA made the interpretation unclear.

Taken together, the FTIR and ssNMR results suggest that pre-processing PVPK30 into the polymer alloy modestly and negatively alters PVPK30’s ability to engage in hydrogen bonding with IVA. Previously, the dissolution performance of ASDs comprising indomethacin (hydrogen bond donor through a carboxylic group) or an esterified indomethacin (unable to act as a hydrogen bond donor) and PVPVA was evaluated as a function of drug loading [50]. Unmodified indomethacin contains a carboxylic acid moiety that engages in substantial hydrogen bonding with PVPVA, while the esterified molecule does not. It was found that for this system, the API’s ability to hydrogen bond with PVPVA dramatically reduced the dissolution performance of the ASDs as the drug loading increases. The indomethacin ASDs showed essentially no dissolution benefit starting at a 15% drug loading, while the esterified indomethacin retained a significant benefit up to a 25% drug loading. Counter-intuitively, at least some systems will experience reduced dissolution performance by increasing the intermolecular interactions between an API and a polymer. The change in the hydrogen bonding ability of IVA in this current study is modest compared to the indomethacin example, but the change in the dissolution performance was also comparatively modest. For these reasons, it appears that the hydrogen bonding capacity of these systems likely contributed to the increased dissolution performance of the polymer alloy ASDs relative to the compositionally identical polymer blend.

These results show that, in addition to particle size alterations, intermolecular interactions between polymers and APIs can be altered through pre-processing by KinetiSol compounding. Through careful choice of the polymer composition and ratios, an increase or decrease in hydrogen bonding or other interactions can be achieved in order to increase the miscibility and dissolution performance of an ASD system. More work with additional compositions of polymer alloy, as well as with APIs with differing properties, will be necessary to understand how this tool can improve ASD properties.

### 3.6. Ivacaftor ASDs Exhibit High Stability under Accelerated Conditions

Because of the decrease in hydrogen bonding between PVPK30 and IVA in the polymer alloy (compared to the polymer blend), there was concern that the physical stability of IVA in this ASD might be reduced. It has previously been shown that hydrogen bonding between an API and a polymer can substantially increase the physical stability of ASDs during storage [51]. It was also hypothesized that the substantial hygroscopicity of PVPK30 would adversely affect the formulations containing it due to the fact of water’s plasticizing effect and ability to reduce Tg [52]. For these reasons, the physical stability of each 40% IVA ASD was evaluated under accelerated conditions (40 °C/75% relative humidity) in both open and closed containers.

To ensure that the measurable crystalline IVA in the 40% IVA HPMCAS ASD prepared by KinetiSol compounding did not confound results, a spray-dried 40% IVA HPMCAS formulation was utilized instead that was amorphous by mDSC and PXRD. This decision was made to reduce the possibility that the residual crystalline material would act as seed crystals and increase recrystallization. As the Tg of IVA has been reported as 175 °C, it was expected that, even under accelerated conditions, the recrystallization would occur over a relatively long period of time [32]. In addition, previous physical stability studies of 50–90% IVA in HPMCAS ASDs have shown that these ASDs are stable under the chosen accelerated conditions for at least 8 weeks [32]. Because of this, a 6-month study period was selected. Each sample (open and closed) was evaluated by PXRD at regular time points for the presence of crystalline material.

**Figure 9:**
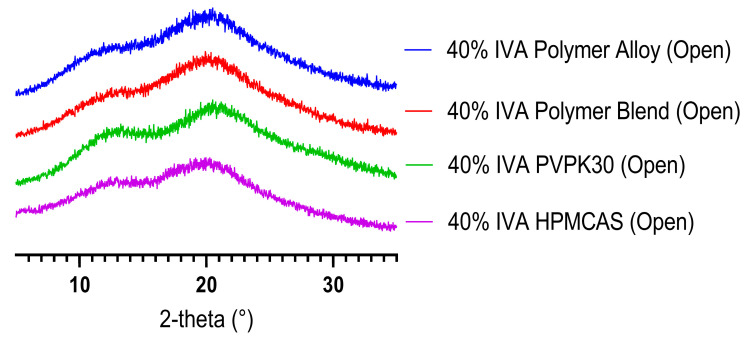
PXRD of 40% IVA ASDs after 6 months in an open container under accelerated stability conditions (40 °C/75%RH). Note that the 40% IVA HPMCAS ASD was spray-dried to avoid the residual crystallinity seen in the KinetiSol ASD.

The PXRD results at all time-points from 1 day to 6 months indicated that the 40% ASDs with either neat polymer, polymer blend, or polymer alloy were amorphous. PXRD data from the 6-month time point are shown in Figure 9. These results suggest that any potential decrease in physical stability due to the fact of either decreased API–polymer hydrogen bonding in the polymer alloy ASD or increased hygroscopicity of the PVPK30 ASD, does not materially affect the physical stability of these systems. The results also indicate that all studied 40% ASDs have acceptable physical stability. As the Tg of an ASD system has been shown to be essential to its physical stability [53], the ability of KinetiSol compounding to alter the Tg of polymers by forming single-phase systems containing a high molecular weight (and a high Tg) PVP could be a substantial benefit for increasing stability. While the systems studied here all had adequate stability, future work should be conducted to understand how polymer choice and polymer pre-processing can affect less stable systems (e.g., those with APIs with a low Tg).

## 4. Conclusions

The composition of an amorphous solid dispersion can affect its maximum drug loading, dissolution performance, physicochemical stability, and bioavailability. This study examined how the creation of a multipolymer, single-phase polymer alloy (pre-processed as an equal mixture of HPMCAS and PVPK30 with 1% SLS) using KinetiSol compounding can alter intermolecular interactions between polymers and IVA to increase the dissolution of 40% IVA ASDs relative to a compositionally identical polymer blend ASD. In addition, the polymer alloy ASD overcame the limitations of each constituent polymer. The DVS results showed that the polymer alloy ASD had reduced hygroscopicity relative to the PVPK30 ASD without the comparatively lowered dissolution performance of the HPMCAS ASD. We also report the processing of a high drug 70% IVA PVPK30 ASD by KinetiSol that was amorphous by mDSC and PXRD. This was possible due to the pre-processing of PVPK30 into larger particles by the polymer alloy method. These studies show the potential benefits that the pre-processing of polymers into polymer alloys can have in the optimization of ASDs. A major limitation of this study is that it addressed only one composition of polymer alloy where each constituent polymer was miscible with the other. Future studies into other compositions of polymer alloys are necessary to better understand the proper use of the polymer alloy method in ASD systems. The examination and use of immiscible polymers, as well as other miscible polymer combinations and ratios, to aid in ASD formulation are promising future research directions.

## Figures and Tables

**Table 1:** Compositions of each formulation processed by KinetiSol compounding. Polymer alloy refers to a mixture of HPMCAS LMP, PVPK30, and sodium lauryl sulfate (SLS) processed into a single-phase mixture by KinetiSol; “-” indicates that this component was not present, except for as a component of polymer alloy, as appropriate.

Formulation	Composition (% *w*/*w*)				
	IVA	HPMCAS LMP	PVPK30	SLS	Polymer Alloy
Polymer Alloy	-	49.5	49.5	1	-
20% IVA HPMCAS	20	79.5	-	0.5	-
20% IVA PVPK30	20	-	79.5	0.5	-
20% IVA Polymer Blend	20	39.75	39.75	0.5	-
20% IVA Polymer Alloy	20	-	-	-	80
40% IVA HPMCAS	40	59.5	-	0.5	-
40% IVA PVPK30	40	-	59.5	0.5	-
40% IVA Polymer Blend	40	29.75	29.75	0.5	-
40% IVA Polymer Alloy	40	-	-	-	60
50% IVA HPMCAS	50	49.5	-	0.5	-
50% IVA PVPK30	50	-	49.5	0.5	-
50% IVA Polymer Blend	50	24.75	24.75	0.5	-
50% IVA Polymer Alloy	50	-	-	-	50

**Table 2:** Gradient high-performance liquid chromatography (HPLC) method for detecting degradant products. Mobile phase A is 0.01N ammonium acetate buffer, and phase B is methanol.

Time (min)	Mobile Phase A (%)	Mobile Phase B (%)
0.0	75	25
6.0	75	25
24.0	30	70
36.0	10	90
42.0	10	90
43.0	75	25
48.0	75	25

**Table 3:** Particle sizes of polymer alloy milled either by tube-milling or cryo-milling.

Identity	Polymer Alloy (Tube-Milled)	Polymer Alloy (Cryo-Milled)
D10 (µm)	31.04	1.12
D50 (µm)	112.57	10.09
D90 (µm)	166.83	54.71

## Data Availability

Data available upon request due to the fact of privacy restrictions.

## Supplementary Materials

The following supporting information can be downloaded at: https://www.mdpi.com/article/10.3390/biomedicines11051281/s1, Figure S1: mDSC of pure ivacaftor; Figure S2: PXRD and mDSC of 70% ivacaftor PVPK30 ASD; Figure S3: ^13^C ssNMR of 20% IVA polymer alloy and 20% IVA polymer blend ASDs; Table S1: Compositions of tablets containing 150 mg ivacaftor for dissolution.
