# Supplementary material for: Pre-Processing a Polymer Blend into a Polymer Alloy by KinetiSol Enables Increased Ivacaftor Amorphous Solid Dispersion Drug Loading and Dissolution
Source: Biomedicines. 2023 Apr 26;11(5):1281. doi: 10.3390/biomedicines11051281 (PMC10215938; doi:10.3390/biomedicines11051281)
Supplement: Supplementary file 1 [file biomedicines-11-01281-s001.zip › biomedicines-2334240-supplementary.pdf]

1. Supplemental Information

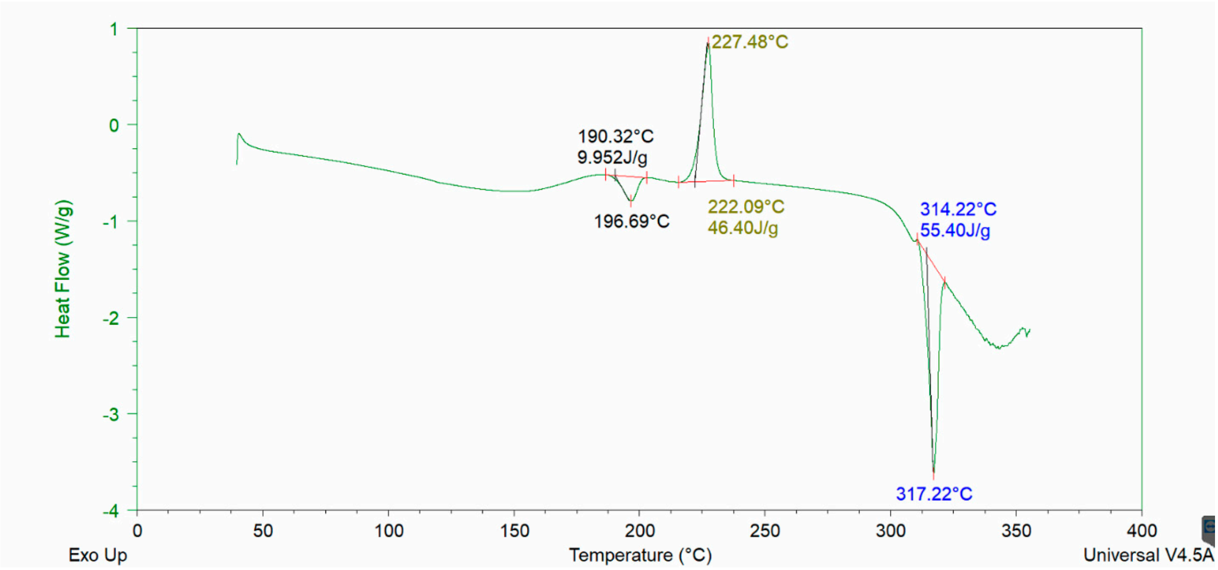

Supplemental Figure S1: mDSC of pure IVA showing two endothermic events: one event at 196.7 ºC and one event associated with the melting of IVA at 317.2 ºC, as well as one exothermic event at 227.5 ºC that is potentially associated with a polymorph change.

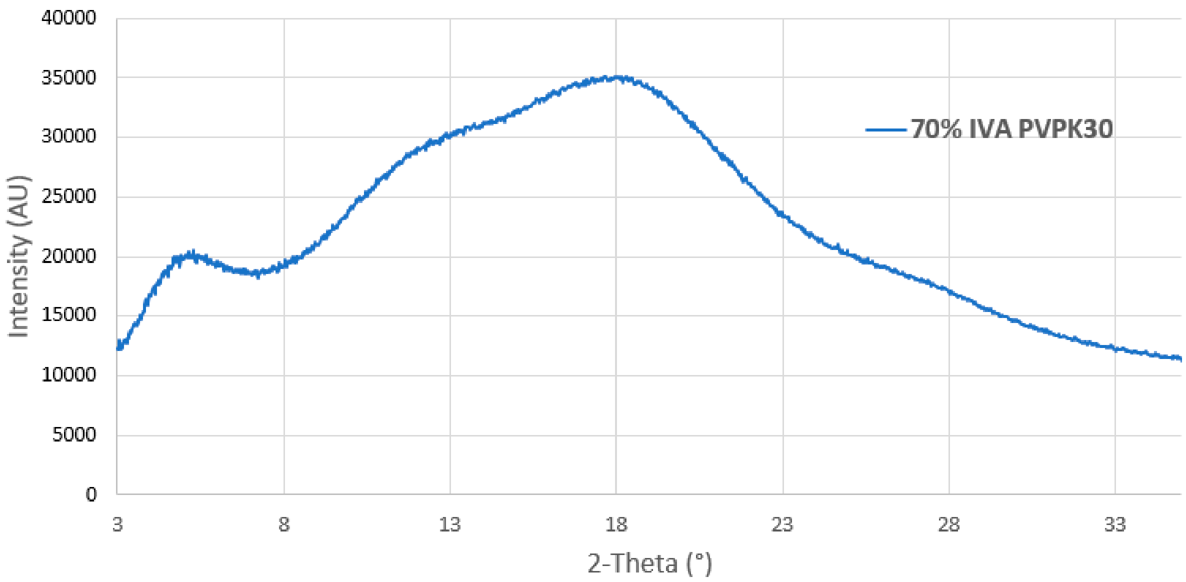

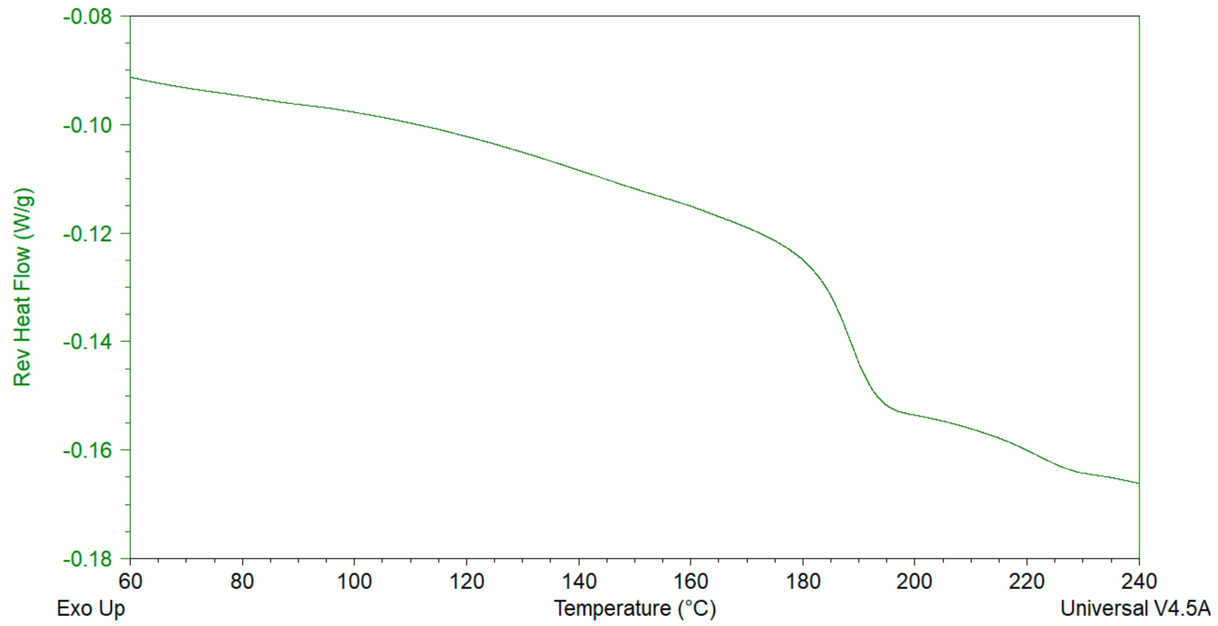

Supplemental Figure S2. PXRD and mDSC of 70% IVA PVPK30 ASD prepared by KinetiSol using PVPK30 that had been pre-processed, milled, and sieved to have a particle size range of 500 µm -1200 µm.

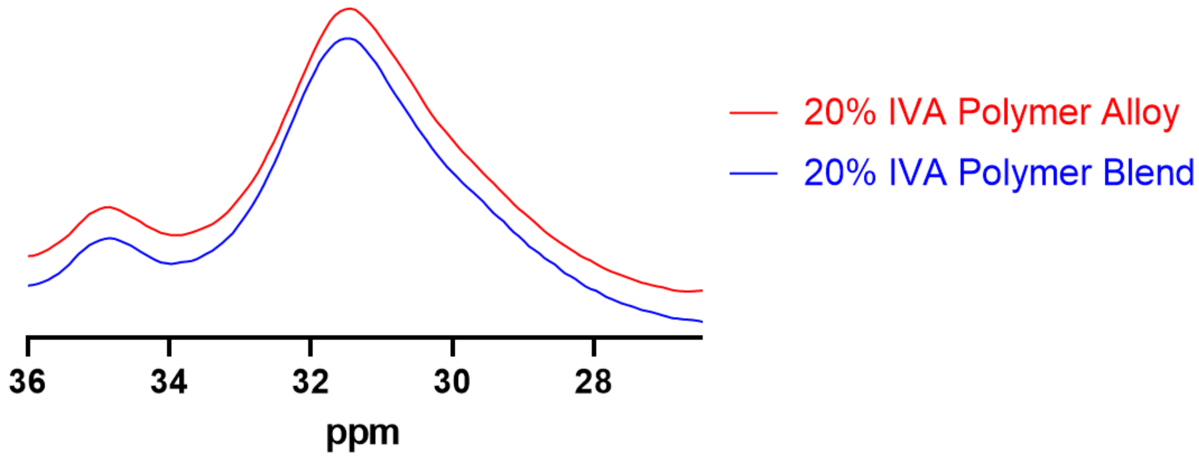

Supplemental Figure S3: ^{13}C ssNMR of 20% IVA polymer alloy and 20% IVA polymer blend ASDs. It should be noted that the peak at 31.44 ppm is comprised of overlapping carbons from PVP (backbone methylene carbon) and IVA (methyl carbons of tert-butyl groups). Units are arbitrary and spectra are overlayed for visual clarity.

Supplemental Table S1: Compositions of tablets containing 150 mg IVA used in dissolution studies.

ASD Drug Load	20% IVA	40% IVA	80% IVA
Percent w/w ASD	60%		
Mass of ASD per 150 mg dose	750 mg	375 mg	187.5 mg
Mass NaCl (10%)	125 mg	62.5 mg	31.25 mg
Mass Na2SO4 (10%)	125 mg	62.5 mg	31.25 mg
Mass Microcrystalline Cellulose (10%)	125 mg	62.5 mg	31.25 mg
Mass croscarmellose	120 mg	60 mg	30 mg
Mass SLS	5 mg	2.5 mg	1 mg
Total tablet mass	1250 mg	625 mg	312.5 mg
